# Coptisine from *Rhizoma Coptidis* Suppresses HCT-116 Cells-related Tumor Growth *in vitro* and *in vivo*

**DOI:** 10.1038/srep38524

**Published:** 2017-02-06

**Authors:** Tao Huang, Yubo Xiao, Lin Yi, Ling Li, Meimei Wang, Cheng Tian, Hang Ma, Kai He, Yue Wang, Bing Han, Xiaoli Ye, Xuegang Li

**Affiliations:** 1 School of Chinese Traditional Medicine, School of Pharmaceutical Sciences, Southwest University, Chongqing 400716, China; 2 Department of Clinical Laboratory, Hunan University of Medicine, Hunan 418000, China; 3 Chongqing Key Laboratory of Translational Research for Cancer Metastasis and Individualized Treatment, Chongqing Cancer Institute & Hospital & Cancer Center, Chongqing 400030, China; 4 School of Life Sciences, Southwest University, Chongqing 400715, China; 5 Chongqing Engineering Research Center for Pharmaceutical Process and Quality Control, Chongqing 400716, China

## Abstract

Colorectal cancer is one of the most common causes of cancer-related death in humans. Coptisine (COP) is a natural alkaloid from *Coptidis Rhizoma* with unclear antitumor mechanism. Human colon cancer cells (HCT-116) and xenograft mice were used to systematically explore the anti-tumor activity of COP in this study. The results indicated that COP exhibited remarkably cytotoxic activities against the HCT-116 cells by inducing G_1_-phase cell cycle arrest and increasing apoptosis, and preferentially inhibited the survival pathway and induced the activation of caspase proteases family of HCT-116 cells. Experimental results on male BALB/c nude mice confirmed that orally administration of COP at high-dose (150 mg/kg) could suppress tumor growth, and may reduce cancer metastasis risk by inhibiting the RAS-ERK pathway *in vivo*. Taken together, the results suggested that COP may be potential as a novel anti-tumor candidate in the HCT-116 cells-related colon cancer, further studies are still needed to suggest COP for the further use.

Colorectal cancer (CRC) is the third leading cause of cancer-related deaths around the world^1^. Early stages of colon cancer are characterized by good prognosis, but once metastasized, the prognosis becomes bad and the 5-year survival rate is only 10%^2^. Therefore, the prevention of tumor growth and metastasis could improve the prognosis of majority of colon cancers.

Recent studies show that several pathways are involved in the initiation and progression of CRC. It has been proven that the mitogen-activated protein kinases (MAPKs) pathways are related to the progress of CRC, which mainly contain three groups: extracellular signal-regulated kinase (ERK), c-Jun N-terminal kinase (JNK) and P38^3^. They are essential for apoptosis and cell proliferation, meanwhile they also regulate the activation and expression of key inflammatory mediators which may function as potent cancer promoters^3^,^4^,^5^. They also play a crucial role in the regulation of cellular adhesion, angiogenesis, invasion, and metastasis in colorectal cancer^6^,^7^,^8^. Furthermore, phosphatidylinositol (PI) 3-kinase-Akt/protein kinase (PI3K-Akt), also known as survival factors, are related to MAPK-ERK1/2 pathways and implicated in cancer progression^9^. These pathways are meaningful for cancer therapy.

Apoptosis, also known as programmed cell death, is activated through two initiating pathways (extrinsic and intrinsic death signal) and a common pathway. It is well known that caspase family serves as the primary mediators of apoptosis. In the extrinsic pathway, cell surface “death receptors” directly activate caspase 8, then the downstream effector caspase 3^10^. In the intrinsic pathway, the BCL-2 family regulate the small molecules such as cytochrome c which leads to the cleavage and activation of caspase 9, and finally caspase 9 also activate the downstream effector caspase 3^11^. Accordingly, we turn our attention to the caspase 8, 9 and their downstream effector caspase 3, which may against with the cancer initiation and progression.

Various MAPK inhibition drugs used in chemotherapy and biologic therapies have been evaluated. For example, salicylates promote apoptosis by the P38-dependent mechanism^12^ and JNK pathway^13^. Drugs targeting ERK signaling pathway lead to substantial improvement in metastatic melanoma, and show a promising activity in additional tumor types^14^. Aspirin plus ABT-737 induce cell death via p38 pathway in human lung cancer^15^. Unfortunately, due to the high drug resistance^16^ or the high incidence of adverse reaction^17^, there is an urgent need for developing more effective preventive agents.

*Rhizoma Coptidis*(RC), the driedrhizome of *Coptis chinensis* Franch., which has a variety of pharmacological effects such as anticachectic^18^, antibacterial^19^, lipid-lowering^20^ and anti-tumor^21^, has been widely used in China. COP, one of main bioactive components in RC, exhibits similar pharmacological activities to RC^22^,^23^. Therefore, it is speculated that COP possibly owns anti-tumor efficiency.

To confirm whether COP exerts anti-tumor activity, here we first investigated the role of COP on the apoptosis and cycle procession in human HCT-116 cells, and further examined the effect of inhibitory action on tumor growth using HCT-116 tumor xenograft mice to figure out the underlying risks of lung metastasis.

## Results

### COP inhibited proliferative in HCT-116 cells and FHC

To seek the effectiveness of COP treatment for HCT-116 cells *in vitro*, cytotoxicity and proliferation were determined by MTT assay and trypan blue exclusion assay, respectively. In addition, human colorectal epithelial cells (FHC) was also used to determine the cytotoxic effect of COP in normal cells.

**Figure 1:**
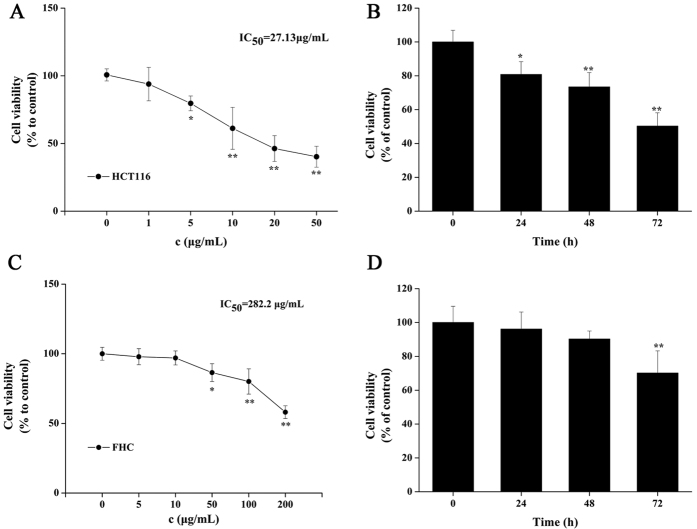
COP induced differential cell death in HCT-116 cells and FHC. (**A**) HCT-116 cells and (**C**) FHC were treated with indicated concentrations of COP for 24 h and cell viability was determined by MTT assay. (**B**) HCT-116 cells and (**D**) FHC were treated with 2.5 μg/mL of COP or PBS for 0 to72 h, and counted using a hemocytometer. Values represent mean ± SD with three replicates. *p < 0.05 and **p < 0.01 compared with control.

As shown in Fig. 1A, in HCT-116 cells, COP exhibited cell cytotoxicity at a high rate (IC_50_ was 27.13 μg/mL vs 282.2 μg/mL in FHC). Besides, COP (2.5 μg/mL) decreased the survival rate of cells time-dependently (Fig. 1B). However, COP treatment induced relatively low cytotoxicity in FHC compared to that in HCT-116 cells (Fig. 1C,D). Based on the results, we chose the optimum concentration range (1–10 μg/mL) and treatment time (24 h) for further *in vitro* studies.

### COP induced apoptosis in HCT-116 cells

**Figure 2:**
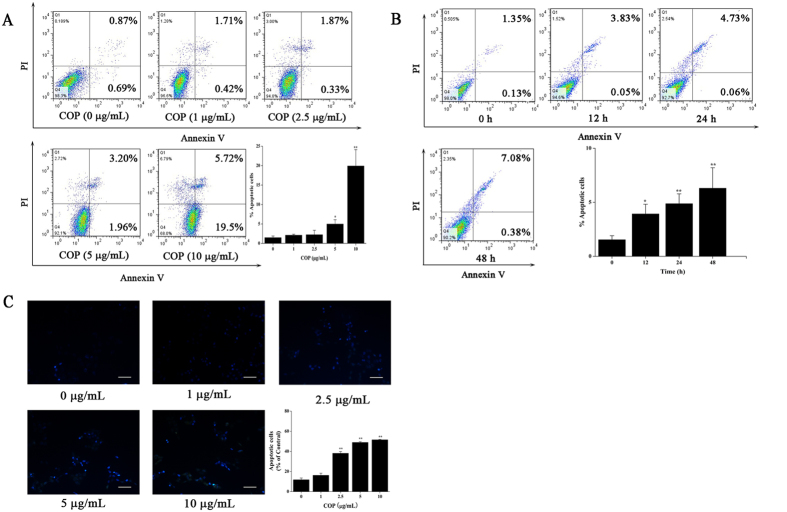
Apoptosis occurred in HCT116 cells after COP treatment. Apoptotic cells were quantified by flow cytometry after stained with Annexin V and PI. (**A**) Cells were treated with indicated concentrations of COP for 24 h. (**B**) Cells were treated with 5 μg/mL of COP for 0 to 48 h. (**C**) Morphologic change of apoptotic cells was evaluated by Hoechst 33342 staining. The scale bar was 50 μm. Values represent mean ± SD with three replicates. *p < 0.05 and **p < 0.01 compared with control.

The result in Fig. 2A showed that a significantly increased apoptosis was induced by COP. After 24 h treatment, the percentage of apoptotic cells was increased from 1.50 ± 0.0712% to 20.9 ± 4.19%. Additionally, we investigated the apoptosis of HCT-116 cells with 5 μg/mL COP at 12 h, 24 h and 48 h. The percentage of apoptotic cells was increased markedly from 1.54 ± 0.37% to 6.31 ± 1.89%, while there was no significant difference between 24 h and 48 h treatment (Fig. 2B). Ulteriorly, Hoechst 33342 staining was used to determine the morphologic change of apoptotic cells. As shown in Fig. 2C, COP treatment in three dosages exhibited much more cells with condensed and fragmented nuclei as compared to the control. We also measured the effect of COP on normal cells (FHC) using flow cytometry. The result shows that after treated with 100 μg/mL of COP, the percentage raised obviously, which is twenty times higher than the one of HCT-116 cells. Therefore, these results suggest that COP is a safe compound for normal cells, and might target some cancer cells specifically. It means that we could ignore the effect of COP on FHC (see Supplementary Fig. S1).

### Effect of COP on HCT-116 cells cycle progression

**Figure 3:**
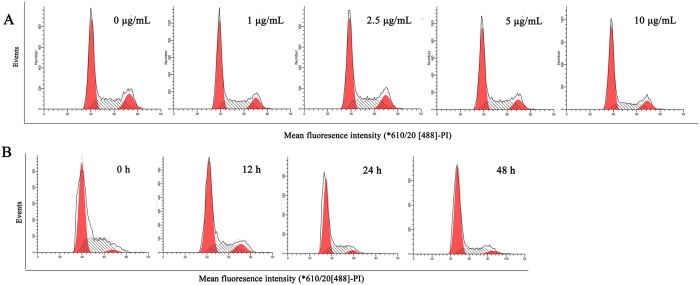
Effect of COP on HCT116 cell cycle progression. Cells were stained with PI solution and analyzed using a flow cytometer. (**A**) Cells were treated with indicated concentrations of COP for 24 h. (**B**) Cells were treated with 5 μg/mL of COP for 0 to 48 h. Values represent mean ± SD with three replicates. *p < 0.05 and **p < 0.01 compared with control.

Accumulation of HCT-116 cells at the G_0_/G_1_-phase occurred in a concentration-dependent after adding of 1–10 μg/mL COP and a time-dependent after treating with 5 μg/mL COP at 12 h, 24 h and 48 h. As shown in Fig. 3A, the percentages of COP-induced cells in the G_0_/G_1_-phase increased from 54.4 ± 1.14% to 62.5 ± 1.13%. Meanwhile, the percentages of the S-phase in the cells treated with stated doses of COP decreased from 29.9 ± 0.75% to 24.6 ± 0.127% (Table 1). Besides, Fig. 3B indicated that the percentages of COP-induced cells in the G_0_/G_1_-phase, after 24 h treatment, increased signally from 60.3 ± 1.54% to 72.0 ± 1.26%, with the percentages of the S-phase decreased from 36.0 ± 0.742% to 23.3 ± 1.05%. But there was no significant difference between 24 h and 48 h treatment (Table 2).

As a result, we chose the treatment time (24 h) for further *in vitro* studies.

### COP induced apoptosis by the extrinsic pathway

**Figure 4:**
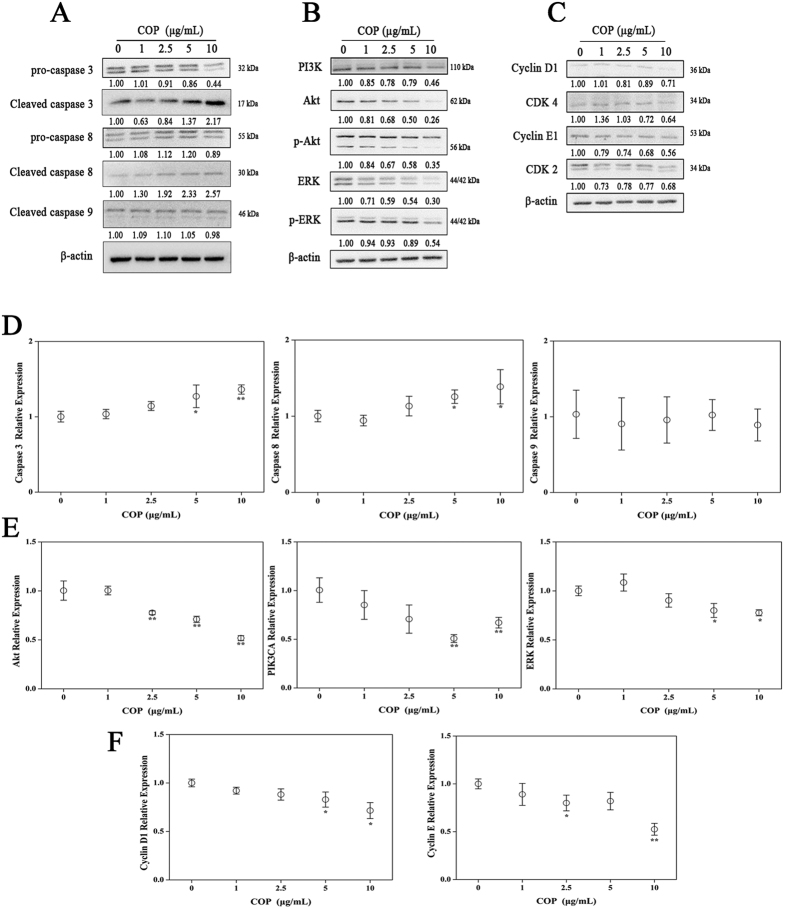
Effect of COP on HCT-116 apoptosis, cycle and survival related proteins and mRNA. Cells were treated with indicated concentrations of COP for 24 h. Then, expression of proteins was determined by Western blotting. The blots were a representative of three independent experiments. (**A**) pro-caspase 3 and 8, cleaved caspase 3, 8 and 9. (**B**) PI3K, ERK, p-ERK, Akt and p-Akt. (**C**) Cyclin D1, Cyclin E1, CDK 4 and CDK 2. The mRNA level of marker gene was assessed by qRT-PCR. (**D**) Caspase 3, 8 and 9. (**D**) Akt, ERK and PIK3CA. (**F**) Cyclin D1 and Cyclin E. Values represent mean percentage ± SD of three replicates. *p < 0.05 and **p < 0.01 compared with control.

The effect of COP on protein levels of the apoptosis markers caspase family was detected as the previous study^10^. As shown in Fig. 4A,D, COP dose-dependently induced the apoptosis by up-regulating the expression of the cleaved caspase 3 and cleaved caspase 8 both in the transcription level and protein expression level. Meanwhile, the expression of pro-caspase 3 decreased proportionately. However, there was no observable change in those of cleaved caspase 9 and pro-caspase 8 (see Supplementary Fig. S2).

### COP blockaded the survival signal pathways of HCT-116 cells

We explored whether the COP could inhibit the cell survival and growth of HCT-116 cells by the PI3-kinase-Akt pathway and the ERK pathway. Treatment on HCT-116 cells with stated doses of COP for 24 h down-regulated the transcription and translation expression of PI3K, AKT and ERK in a concentration-dependent manner (Fig. 4B,E and Supplementary Fig. S3).

### COP blockaded the G_0_/G_1_-phaseregulatory pathways of HCT-116 cells

The effect of COP on cell cycle-regulatory genes specific for the G_0_/G_1_-phase was then determined. COP significantly decreased protein levels of CyclinD1, Cyclin E and their compounds (CDK 4 and CDK 2) in a concentration-dependent manner compared to the control (Fig. 4C and Supplementary Fig. S4). The mRNA level of CyclinD1 and Cyclin E were also declined markedly (Fig. 4F).

### Effect of COP on organ coefficient of nude mice

To investigate whether COP effected the growth of nude mice in the presence or absence of tumor cells, the organ coefficients were recorded. In the absence of tumor cells, there was no influence on the organ coefficients without or with the COP treatment (Table 3). It was in accord with the previous results on antihypercholesterolemic effect of COP^24^. While the mice were inoculated with tumor cells, the organ coefficients were significantly reduced compare with the NC group, including liver, lung and spleen. Oppositely, there was not markedly different among the TC group and COP treatment groups (Table 3). A dose of 150 mg/kg of COP was determined to be the low toxicity doses. These data suggested that coptisine was safe which could be used for further *in vivo* study.

### COP decreased xenograft tumor growth

**Figure 5:**
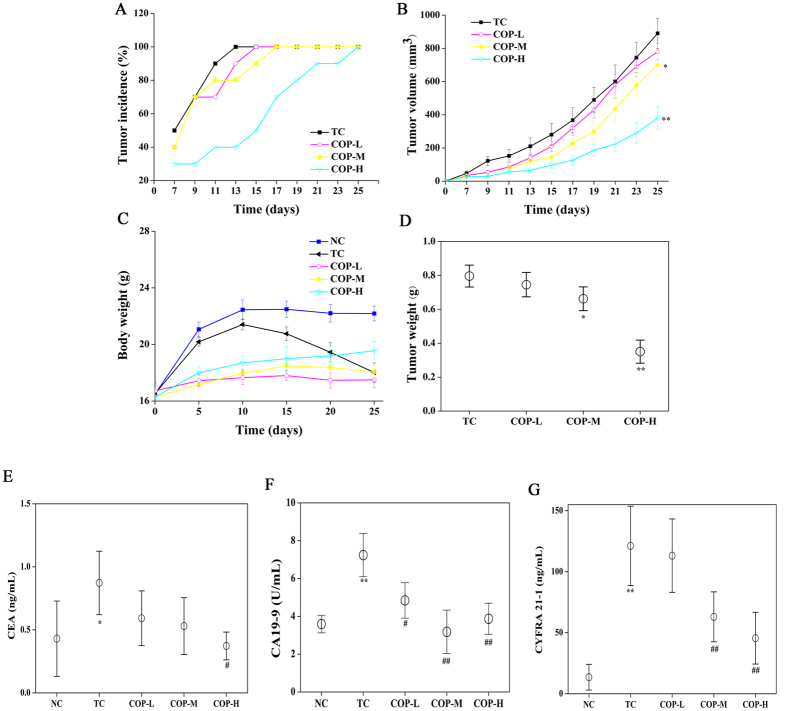
Effect of COP on tumor growth in human tumor xenograft mice. HCT-116 cells were injected intradermally into the flank of BALB/c-nude mice separately, meanwhile COP was administered orally to mice every day for 25 days. At the end of experiment, the serum of mice was obtained and analyzed using multi-tumor markers protein chip diagnose system. (**A**) Tumor incidence. (**B**) Tumor volume was measured and calculated every two days. (**C**) Body weight was measured every five days. (**D**) Tumor weight was recorded at the end of the study. (**E**) CEA. (**F**) CA19-9. (**G**) CYFRA 21-1. Values represent mean ± SD. n = 10, *p < 0.05 and **p < 0.01 compared with NC group, ^#^p < 0.05, ^##^p < 0.01 compared with TC group. NC represented negative control; TC represented tumor control. COP-L: Coptisine at low dosage (50 mg/kg); COP-M: Coptisine at medium dosage (100 mg/kg); COP-H: Coptisine at high dosage (150 mg/kg).

When injected to the nude mice, the HCT-116 cells grew in local rapidly and produced distinguishably large tumors as shown in Supplementary Fig. S5. Treatment was begun with COP by gavage on the day of inoculation. On day 7, tumor initiation could be recorded. As shown in Fig. 5A, tumor occurrence was significantly delayed in all the treatment groups as compared with the tumor control (TC) group. On day 25, the tumor volume of COP-H groups was significantly smaller than tumors in the TC group (380.3 ± 134.7 mm^3^ vs. 890.9 ± 122.9 mm^3^, P < 0.01) (Fig. 5B).

As shown in the Fig. 5C, once the tumor was formed, the weight of TC group gradually reduced with the increasing of the tumor. Although COP-H could increase the body weight as compared to TC group, it was still below to the negative control (NC) group. The other groups showed no significant changes. More importantly, COP reduced the weight of the tumor dose-dependently. Especially, COP at a medium and high dosage decreased the tumor weight to 0.663 ± 0.121 g (P < 0.05) and 0.351 ± 0.220 g (P < 0.01) from 0.803 ± 0.191 g of TC group (Fig. 5D).

The serum tumor markers, CEA and CA199, were measured to diagnose the occurrence and prognosis of colon cancer. CYFRA 21-1 is a polypeptide tumor marker, whose diagnostic utility and prognostic relevance have been demonstrated in non-small-cell lung cancer (NSCLC) or colorectal cancer^25^,^26^. As shown in Fig. 5E–G, when the mice were inoculated with tumor cells, the levels of CEA, CA19-9 and CYFRA 21-1 in the TC group were significantly higher than those of the NC group. The administration of COP could reverse the trend of the three serum markers in a dose-dependent manner. COP with the high dosage exhibited the best activity, which decreased the levels of CEA, CA19-9 and CYFRA 21-1by 54.1%, 42.6% and 58.3% compare to TC group respectively (P < 0.01).

These indicated that COP can effectively interfere with the progression of colon cancer.

### COP affected tumor and lung MAPK pathway expression

**Figure 6:**
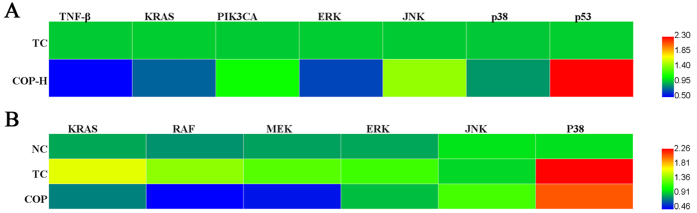
Levels of MAPK pathways genes expression were assessed by qRT-PCR. The xenograft mice were administered with indicated concentrations of COP for 25 days. At the end of experiment, the tumors and lungs were taken and used to extract RNA. The hot map was a representative of three independent experiments. (**A**) The expression of TNF-β, KRAS, PIK3CA, ERK, JNK, p38 and p53 in tumors. (**B**) The expression of KRAS, RAF, MEK, ERK, JNK and p38 in lungs.

The qRT-PCR experiments were performed on lung and tumor tissue samples from tumor-bearing mice. The expression of TNF-β, KRAS, PIK3CA, ERK, JNK, p38 and p53 in tumors were analyzed. As shown in Fig. 6A, high-dose COP reduced the oncogene activation markedly, such as TNF-β and KRAS, enhanced the tumor suppressor gene p53 signally and affected the MAPK pathway, especially ERK (see Supplementary Fig. S6).

In the lungs, the effect of high-dose COP on ERK, JNK and P38, was detected (Fig. 6B). The results indicated that COP significantly reduced in mRNA level of ERK, but not JNK or P38. Subsequently, we determined the effect of COP on upstream modulation of ERK at the transcriptional level. The qRT-PCR data showed that the mRNA expression of MEK, RAF and KRAS were significantly increased after inoculation when compared to the NC group, whereas they were significantly down-regulated after the COP treatment (see Supplementary Fig. S7).

## Discussion

The current research indicates that isoquinoline alkaloids and their N-oxides exhibited widest spectra of pharmacological activities. There may be a potential structure-activity relationship among them^27^. Coptisine (COP), a natural alkaloid, also owns similar structure of isoquinoline alkaloids and possibly exhibits an anti-tumor property.

In this work, molecular mechanisms of COP-induced HCT-116 growth and proliferation were studied. We focused mainly on roles of apoptosis and cell cycle arrest by COP. The results of Annexin V/PI and Hoechst 33342 staining showed that COP could induce apoptosis in HCT-116 cells. The effect of COP on the transcriptional and translational levels of key genes suggested that caspase-dependent apoptotic pathways were triggered in COP-treated HCT-116 cells. Additionally, we found that COP exhibited significant inhibition of proliferation to human colon cell lines by causing the cycle arrest. Here, when the cells were treated with COP for 24 h, G_0_/G_1_ cell cycle is arrested, Cyclin D1 and Cyclin E decreased significantly. These results suggest that both signalling pathways play important roles in the growth and proliferation of HCT-116 cells treated with COP.

However, it is well known that the PI3K-Akt and MEK-ERK pathways promote cell survival and against apoptosis, the phosphorylation is considered a key factor in the development of cancer^28^. Our results confirmed that COP observably down-regulated protein expression of AKT, ERK and their active forms in a concentration-dependent manner. But the total protein expression is lower than the active form in the presence of COP with the same doses. It may be more inclined to inhibit prototype, and reduce activation form indirectly. It was suggested that inhibition to the survival pathway is related to COP-associated apoptosis in HCT-116 cells. Furthermore, previous studies have shown that ERK activation is important for cell-cycle progression from G_0_/G_1_ to S phase in several ways, including stabilization of c-Myc, induction of the p21 or p27 expression and down-regulation of anti-proliferative genes^29^,^30^. As the key genes of G_1_/S-phase, Cyclin D1 also play a role in ERK activation^31^. Thus, COP-induced inhibition of cell growth might be dependent on ERK pathway.

These data were further confirmed *in vivo* using HCT-116 xenograft mouse mode. Animals receiving HCT-116 cells while treating with COP delayed tumorigenesis and exhibited markedly reduced tumor volume and weight rather than the body weight. According to a mountain of our previous works, we have evaluated safety of main alkaloids (berberine, coptisine, palmatine and epiberberine) from Rhizoma Coptidis by the acute toxicity on mice and the sub-chronic toxicity on rats. Previous study showed that the LD_50_ value of COP was about 880 mg/kg on Kunming mice. There were hardly changes in animal deaths, toxic symptoms in the hematological analysis or in relative tissue weights of the vital organs, during the 90-day administration of coptisine with a dosage of 154 mg/kg·day on SD rats. Moreover, there was no abnormality in clinical signs, organ weights, urinalysis, hematological parameters (such as ALT, AST, GGT), gross necropsy and histopathology in any of the animals after the oral administration during the whole sub-chronic toxicity study period^24^,^32^. Furthermore, COP can decrease weight of the hyperlipidemia mice which caused by high-fat diet, with each organ weight reducing. Importantly, there was no difference in the ratio of organ weight to body weight between COP-treated group and normal control group^32^. Similarly, in this experiment, we also found that the COP treatment did not influence the ratio between organ and body weight of nude mice. It suggests that COP is a low-toxicity compound.

As HCT-116 cells could secrete carcinoembryonic antigen (CEA) into blood, it could be easy measured. CEA is the most routinely used colorectal tumor marker. It has a role in estimating the risk of metastatic potential of colorectal cancers to the liver as a facilitator of the inflammatory response^33^,^34^. It causes activation and production of pro- and anti-inflammatory cytokines including IL-1, IL-10, IL-6 and TNF-α, which has implications for the control of tumor cell implantation and survival in the liver^35^. Carbohydrate antigen 19-9 (CA199) is a marker of variety of gastrointestinal tumors including colorectal cancer, pancreatic cancer and hepatocellular carcinoma. The sensitivity and specificity for colorectal cancer is 32% and 96% respectively^36^,^37^. CYFRA 21-1 test is a useful auxiliary test in the diagnosis of non-small cell lung cancer because of its high sensitivity and specificity, its diagnostic utility and prognostic relevance have been demonstrated in colorectal cancer^26^,^38^. It may be a prognostic factor to monitor the lung metastasis of colon cancer. Here, we also found serum CEA, CA199 and CYFRA 21-1 in xenograft mice is decreased compared to pre-administration of all the dosage regimens. These findings indicated an important role of COP in interfering with the initiation and progress stage of colon cancer.

The study found that COP significantly down-regulated the transcription of both oncogene KRAS and TNF-β in the tumor tissues by MAPK pathway, meanwhile up-regulated the expression of tumor-suppressor genes p53. Among of them, oncogenic RAS mutation is the most frequent mutation in colon cancers, and KRAS is the most commonly mutated isoform of RAS. The RAS gene mutation induces the generation of cancer by stabilizing cascade reaction and activating downstream signaling. The RAS-MAPK pathway which was an evolutionary conserved signaling cascade exactly promote proliferation and metastasis^39^, and the downstream acceptor ERK may play a pivotal role^40^,^41^,^42^. These findings possibly expand our understanding of the role of ERK in the metastasis progression and suggested that it would be an effective therapeutic target. There were many groups which have already started to explore the ERK signaling inhibitors in patients with RAS-mutant tumors^43^. Even though combinatorial treatment or alternating therapy with distinct inhibitors of the same pathway solves the resistance of the single agent therapy in a certain extent, effective inhibition of RAS-ERK signaling was still a major challenge^14^.

Moreover, in the process of human colon cancer, 40% of patients with CRC are diagnosed at a local stage, among them approximately 20% patients with distant metastases^44^. One of the most primary site was liver, followed by the lung^2^. Because of the limitation of cell characteristics, no liver or lung metastases were observed in the control group injected with the HCT-116 cells. Then we used these relatively unaggressive cell lines to evaluate metastatic potential after drug treatment. The fact was that the xenograft model of colon cancer may induce the transcription of both KRAS and ERK of lung tissues, which may reduce the risk of colon cancer metastasis and wish to provide help for further research.

In summary, COP, possibly, prevents the initiation and growth of colon cancer as attest in a xenograft mouse model, primarily via reducing tumor cell proliferation and inducing caspase-dependent apoptosis. However, because of the limitation of animal models, the mechanism of lung metastatic and therapeutic effect of COP is insufficient. All these postulations still need further study.

## Materials and Methods

### Reagents

Coptisine (>98.0% by HPLC) was extracted from RC by an established procedure in our lab (the details were described in Supplementary Methods) and stored in −80 °C before use. Other chemicals were indicated otherwise. The primary antibodies against p44/42 MAPK (ERK 1/2) and P-p44/42 MAPK (ERK 1/2) (T202/Y204) were obtained from Cell Signaling Technology (Boston, USA). The antibody against Cyclin D1, Cyclin E1, CDK 2, CDK 4, PI3K, AKT, pro-caspase 3, cleaved caspase 3, cleaved caspase 9, caspase 8, β-actin and rabbit IgG were obtained from Proteintech (Wuhan, China). The antibody against p-Akt 1/2/3 was obtained from Santa Cruz Biotechnology (California, USA). Other chemicals were indicated otherwise.

### Cell culture

The human HCT-116 cells and FHC were obtained from Cell bank of Chinese academy of sciences and cultured in Dulbecco’s modified Eagle’s medium (DMEM) (Gibco, New Zealand) supplemented with 10% fetal bovine serum (Gibco, New Zealand), 55 mg/mL streptomycin and 55 IU/mL penicillin (Invitrogen, USA) at 37 °C in a humidified atmosphere of 5% CO_2_.

### Cell viability assay

The cells were transferred onto 96-well culture plates at a density of 5000 cells per well. After incubation overnight, the cells were treated with designed concentrations of COP (1 to 50 μg/mL for HCT-116 cells, 1 to 200 μg/mL for FHC) or phosphate buffer saline (PBS) for 20 h at 37 °C. Then MTT reagent (20 μL, 1.5 mg/mL in PBS) was added to each well and incubated for 4 h. The supernatant was removed, and 200 μL of DMSO was added to dissolve the formazan crystals. Absorbance was measured with spectrophotometer at 490 nm wavelength^45^.

### Proliferation assay

HCT-116 cells or FHC were seeded in triplicate onto 12-well plates at a density of 10^5^cells/well and grown for 24 h. Then cells were treated with 2.5 μg/mL of COP or PBS for different time points (24,48 and 72 h). Thereafter, viable cell number was determined by the trypan blue exclusion assay according to the manufacturer’s instructions.

### Flow cytometry

Cellular apoptosis was analyzed using Annexin V and propidium iodide (PI) kit according to the manufacturer’s instructions. To determine the cell cycle assay, the treated cells were harvested, fixed overnight with 70% ethanol at 4 °C and incubated with PI staining reagent^46^. Then the stained cells were subjected to a BD FACSVantage SE Flow Cytometer (BD Biosciences, San Jose, CA, USA). Data were analyzed using Flow Jo 7.6.1 (Tree Star Inc., Ashland, OR, USA) and Modfit (BD Biosciences, USA) software.

### Hoechst 33342 staining

After treatment at 6-well plates for 24 h, as the previously described^45^, cells were stained with dye Hoechst 33342at 37 °C for 5 min and avoided from light. Then, the cells were washed with 1 × PBS and images were captured on a Nikon fluorescence microscope (Nikon, Japan).

### Animals

Four-week-old male BALB/c nude mice (Beijing, China) were housed in pathogen-free room of Southwest University in pressurized ventilated cages according to institutional regulations until the experiment begin. Animal experiments were officially approved by the animal care and use review committee of Southwest University. All animal studies were carried out in accordance with the NIH guidelines in 2011 for the care and use of laboratory animals, including any relevant details.

### Tumor xenograft studies

Animal experiments were carried out as described previously^47^,^48^. To implant the colon tumors, cells (5–6 * 10^6^) were harvested, suspended in serum free medium and inoculated in the right foreleg of per mice (n = 10 mice/group). From the day of inoculation, COP was daily administered in gavage at 50,100 or 150 mg/kg body weight, marked as COP-L, COP-M and COP-H respectively. Tumor xenografts development was followed every two days by Vernier caliper measurement along two orthogonal axes, length (L) and width (W). The volume (V) of it was calculated by the equation for ellipsoid (V = W^2^ * L/2)^49^. Mouse body weight was measured two times per week. At the time of sacrificing (day 25), blood samples were collected for multiple tumor marker protein biochip assay, and tumor xenografts were quickly dissected away from surrounding tissue and weighed. Then, they were divided into two parts and stored at −80 °C for biochemical analyses. Organ weights of heart, liver, spleen, lung and kidneys were weighed in order to monitor any side effect of treatments.

### Serum tumor markers test

The plasma samples were submitted to the Chongqing Cancer Hospital to test the serum tumor markers. Multi-tumor markers protein chip diagnose system was used to detect nine tumor markers in nude mouse colon cancer xenograft and negative control (NC) group, and further analysis was undertaken based on the results^50^.

### Real-time qPCR analysis

RNA was extracted from lung tissues, tumor tissues or cells using Trizol reagent (Thermo Fisher, USA) according to the manufacturer’s instructions (n = 3/group). Samples were processed with chloroform and subsided by isopropyl alcohol. Finally, RNA was dissolved in RNase-free water and quantified. cDNA synthesis was achieved by using the iScript™cDNA synthesis kit (Bio-Rad, CA, USA). Real-time PCR reactions were conducted according to the SsoAdvanced™ Universal SYBR Green (Bio-Rad, CA, USA) protocol and the primers were used as shown in the Table 4. The relative quantitation of gene expression was determined by the ^∆∆^Cq method. To normalize the output for each sample, the expression of genes was divided by GAPDH gene expression.

### Western blot analysis

Total protein was extracted from the cells by 1X RIPA buffer (BBI life sciences, China) containing 1 mM phosphatase inhibitor cocktail and 1 mM phenylmethylsulfonyl fluoride (PMSF). Equal amounts of proteins (10 μg) were subjected to SDS-PAGE. After transferred onto a polyvinylidene fluoride membrane, they were incubated with primary antibodies against PI3K, pro-caspase 3, cleaved caspase 3, pro-caspase 8, cleaved caspase 8, cleaved caspase 9, AKT, β-actin, ERK, p-ERK, Cyclin D1, Cyclin E1, CDK 2, CDK 4 and p-AKT, respectively (1:1000 dilution, 4 °C, overnight). Western blot was quantified by using Image J software (the full blots were provided in Supplementary Material).

### Statistical analysis

All values were expressed as the mean ± standard deviation (SD). In the vitro studies, the treated-groups and control groups were compared by Student’s t-test. In the vivo studies, the differences among each group were analyzed by one-way ANOVA with the Turkey multiple comparison test. The statistical significance of data was P < 0.05.

## Additional Information

**How to cite this article**: Huang, T. *et al*. Coptisine from *Rhizoma Coptidis* Suppresses HCT-116 Cells-related Tumor Growth *in vitro* and *in vivo. Sci. Rep.* **7**, 38524; doi: 10.1038/srep38524 (2017).

**Publisher's note:** Springer Nature remains neutral with regard to jurisdictional claims in published maps and institutional affiliations.

## Supplementary Material

Supplementary Information

## Figures and Tables

**Table 1:** Effect of COP on HCT116 cell cycle progression.

COP (μg/mL)	Cell cycle distribution (%)		
	G_0_/G_1_	S	G_2_/M
0	54.4 ± 1.14	29.9 ± 0.751	15.6 ± 0.404
1	57.1 ± 0.333*	29.7 ± 0.505	13.2 ± 0.325**
2.5	53.0 ± 0.721	32.6 ± 1.03**	14.4 ± 0.604
5	56.5 ± 0.794	32.1 ± 0.350*	11.4 ± 0.648**
10	62.5 ± 1.13**	24.6 ± 0.127**	12.9 ± 1.02**

HCT-116 cells were treated with COP at 1–10 μg/mL for 24 h and harvested by trypsinization. The cells were stained with PI solution, and 10000 events per experiment were analyzed using a flow cytometer. Values represent mean ± SD with three replicates. *p < 0.05 and **p < 0.01 compared with control.

**Table 2:** Effect of COP on HCT116 cell cycle progression.

Time (h)	Cell cycle distribution (%)		
	G_0_/G_1_	S	G_2_/M
0	60.3 ± 1.54	36.0 ± 0.742	3.62 ± 1.59
12	64.8 ± 4.25	27.3 ± 3.54**	7.90 ± 1.27**
24	68.3 ± 1.84*	26.7 ± 1.77**	5.02 ± 0.0800
48	72.0 ± 1.26**	23.3 ± 1.05**	4.68 ± 0.215

HCT-116 cells were treated with COP at 5 μg/mL for 0, 12, 24, 48 h and harvested by trypsinization. The cells were stained with PI solution, and 10000 events per experiment were analyzed using a flow cytometer. Values represent mean ± SD with three replicates. *p < 0.05 and **p < 0.01 compared with control.

**Table 3:** Effects of COP on ratio between organ and body weight (%).

Groups	Tumor Cells injection	Heart (%)	Liver (%)	Spleen (%)	Lungs (%)	Kidneys (%)
NC	−	0.571 ± 0.352	4.61 ± 1.04	0.334 ± 0.190	0.643 ± 0.161	1.61 ± 0.0333
COP-H	−	0.554 ± 0.221	4.71 ± 0.701	0.343 ± 0.132	0.664 ± 0.135	1.62 ± 0.253
TC	+	0.423 ± 0.194*	3.76 ± 1.55**	0.221 ± 0.160**	0.504 ± 0.167*	1.59 ± 0.0434
COP-L	+	0.421 ± 0.163*	3.79 ± 1.20**	0.194 ± 0.161**	0.596 ± 0.253	1.63 ± 0.0981
COP-M	+	0.413 ± 0.220*	3.71 ± 1.14**	0.233 ± 0.223**	0.618 ± 0.851	1.56 ± 0.283
COP-H	+	0.420 ± 0.413*	3.78 ± 2.34**	0.231 ± 0.160**	0.590 ± 0.251	1.61 ± 0.0632

(a) Data were mean ± SD, n = 10. *p < 0.05, **p < 0.01 compare to NC group. (b) NC represented the negative control; TC represented the tumor control. COP-L: Coptisine at low dosage (50 mg/kg); COP-M: Coptisine at medium dosage (100 mg/kg); COP-H: Coptisine at high dosage (150 mg/kg).

**Table 4:** Primer sequences used for quantitative RT-PCR.

Primer name	Species	Forward primer	Reverse primer	Accession number	Product length (bp)
PI3KCA	human	5′-gacgactttgtgaccttcg-3′	5′-gaagtcctgtacttctggat-3′	NM_006218.3	158
Akt1	human	5′-atgagcgacgtggctattgt-3′	5′-tgaaggtgccatcattcttg-3′	NM_005163.2	106
ERK (MAPK1)	human	5′-tgcagatccagaccatgatc-3′	5′-gaatgcagcctacagaccaa-3′	NM_002745.4	134
mice	5′-cgcttcagacatgagaacatc-3′	5′-ggtccgtctccatgaggt-3′	NM_011949.3	106	
Caspase 3	human	5′-ctcggtctggtacagatgtcga-3′	5′-catggctcagaagcacacaaac-3′	NM_004346.3	177
Caspase 9	human	5′-gtggacattggttctggaggat-3′	5′-cgcaacttctcacagtcgatg-3′	NM_001228.4	188
Caspase 8	human	5′-ggtcacttgaaccttgggaa-3′	5′-aggccagatcttcactgtcc-3′	NM_001229.4	114
Cyclin D1 (CCND1)	human	5′-cacgcttcctctccagagtg-3′	5′-ccaggttccacttgagcttgt-3′	NM_053056.2	170
JNK (MAPK8)	mice	5′-tcccagctgactcagagcat-3′	5′-gcttcatctacggagatcctt-3′	NM_016700.4	106
P38 (MAPK14)	mice	5′-cccagcaacctagctgtg-3′	5′-gctcggtaccacctggtag-3′	NM_011951.3	113
MEK1 (MAP2K1)	mice	5′-tgggcacgagatcctacatg-3′	5′-tggcatcaggaggaggaatg-3′	NM_008927.3	135
KRAS	mice	5′-gcctgctgaaaatgactgagt-3′	5′-cttgacctgctgtgtcgaga-3′	NM_021284.6	19
RAF1	mice	5′-tcgatgtcagacttgtggctac-3′	5′-tctcgcatccgacgcattg-3′	NM_029780.3	168
GAPDH	human	5′-gaaggtgaaggtcggagtca-3′	5′- ttgaggtcaatgaaggggtc-3′	NM_002046.5	117
mice	5′-cgaccactttgtcaagctca-3′	5′- aggggtctacatggcaactg-3′	NM_008084.3	223	
