# Supplementary material for: Coptisine from Rhizoma Coptidis Suppresses HCT-116 Cells-related Tumor Growth in vitro and in vivo
Source: Sci Rep. 2017 Feb 6;7:38524. doi: 10.1038/srep38524 (PMC5292956; doi:10.1038/srep38524)
Supplement: Supplementary Information [file srep38524-s1.pdf]

Coptisine from *Rhizoma Coptidis* Suppresses HCT-116 Cells-related Tumor Growth *in vitro* and *in vivo*

Tao Huang[a*], Yubo Xiao[a,e*], Lin Yi[b], Ling Li[a], Meimei Wang[a], Cheng Tian[a], Hang Ma[a], Kai He[a,e], Yue Wang[c], Bing Han[a], Xuegang Li[a,d**], Xiaoli Ye[c,d**]

[a] School of Chinese Traditional Medicine, School of Pharmaceutical Sciences, Southwest University, Chongqing, 400716, China

[b] Chongqing Key Laboratory of Translational Research for Cancer Metastasis and Individualized Treatment, Chongqing Cancer Institute, Chongqing, 400030, China

[c] School of Life Sciences, Southwest University, Chongqing, 400715, China

[d] Chongqing Engineering Research Center for Pharmaceutical Process and Quality Control, Chongqing, 400716, China

[e] Department of Clinical Laboratory, Hunan University of Medicine, Hunan, 418000, China

[*]These authors contributed equally to this work and should be considered co-first authors

[**]Corresponding author: X.G. Li (Please address all letters to this author)

Tel: +86 023-68250728; Fax: +86 023-68250728.

E-mail addresses: xuegangli@swu.edu.cn

Address: School of Pharmaceutical Sciences, Southwest University, No.2 Tiansheng Road, Beibei District, Chongqing, 400715, China

X.L. Ye

Tel: +86 023-68250728; Fax: +86 023-68250728.

E-mail addresses: yexiaoli@swu.edu.cn

Address: School of Life Sciences, Southwest University, No.2 Tiansheng Road, Beibei District, Chongqing, 400715, China

Supplementary Methods

Extraction of COP from Rhizoma *Coptidis*

Raw materials of Rhizoma *Coptidis* (1000 g) were extracted by 1 L ethanol under room temperature for 24 h, extracted 3 times with same method. Then, the supernatant was collected and evaporated to dryness under rotary evaporators to obtain the ethanol extract (300 g). It was separated by high speed counter current chromatography (HSCCC). Solvent system was CHCl3-MeOH-0.2M HCl (4:2:2, v/v/v), mobile phase was lower organic phase. The column temperature was set at 25 ℃, revolution 800 rpm/min, and mobile phase flow rate 2.0 ml/min. Peak fractions were collected according to the elution profiles. The fourth peak were collected in one run and then purified by sephadex LH-20 column to obtain the coptisine (21mg).

Supplementary Material

Full blots

pro-caspase 3 (0,0,1,2.5,5,10 μg/mL COP)

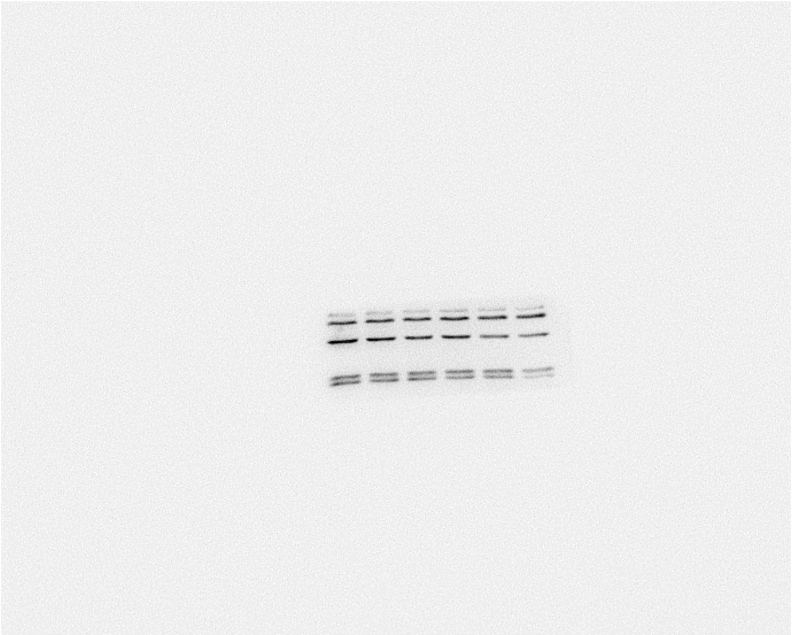

Cleaved caspase 3 (0,1,2.5,5,10,20 μg/mL COP)

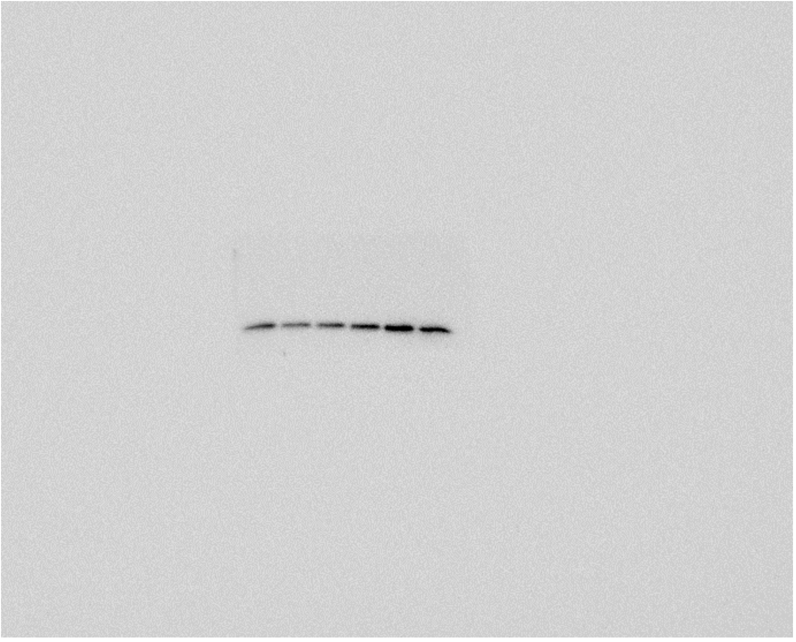

Pro-caspase 8 (0,0,1,2.5,5,10 μg/mL COP)

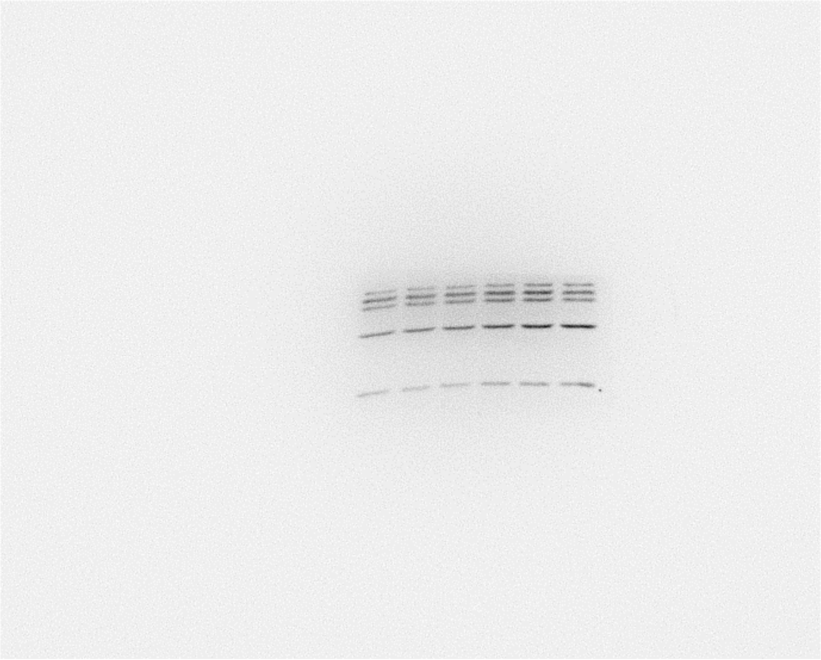

Cleaved caspase 8 (0,0,1,2.5,5,10 μg/mL COP)

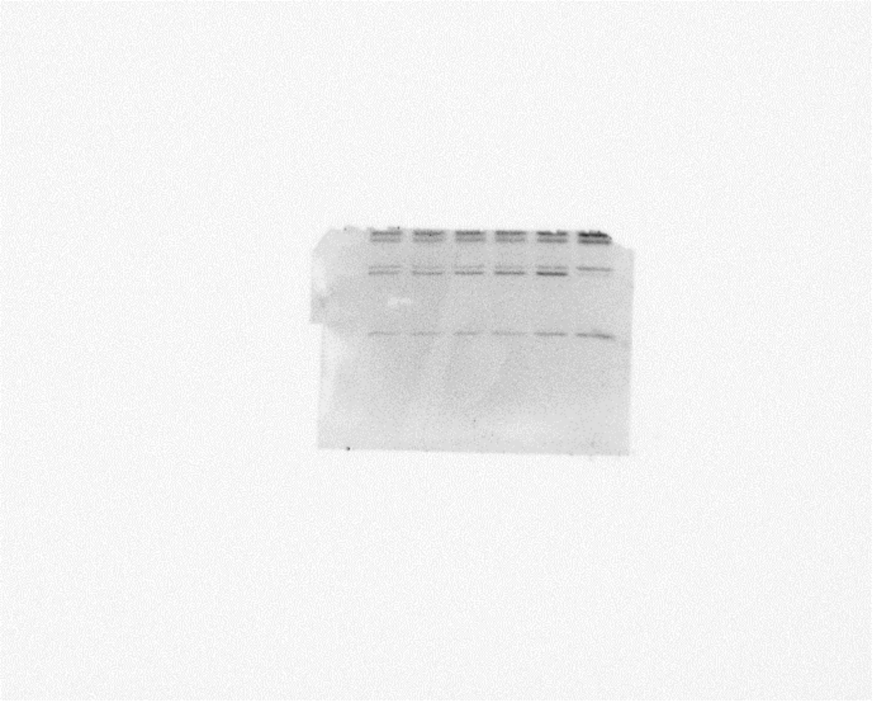

β-actin (0,0,1,2.5,5,10 μg/mL COP)

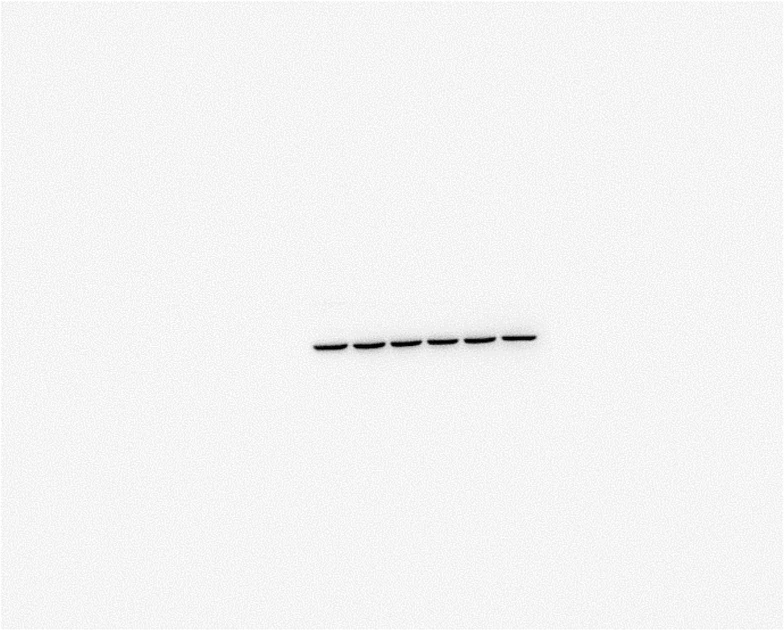

Cleaved caspase 9   (0,1,2.5,5,10,20 μg/mL COP)

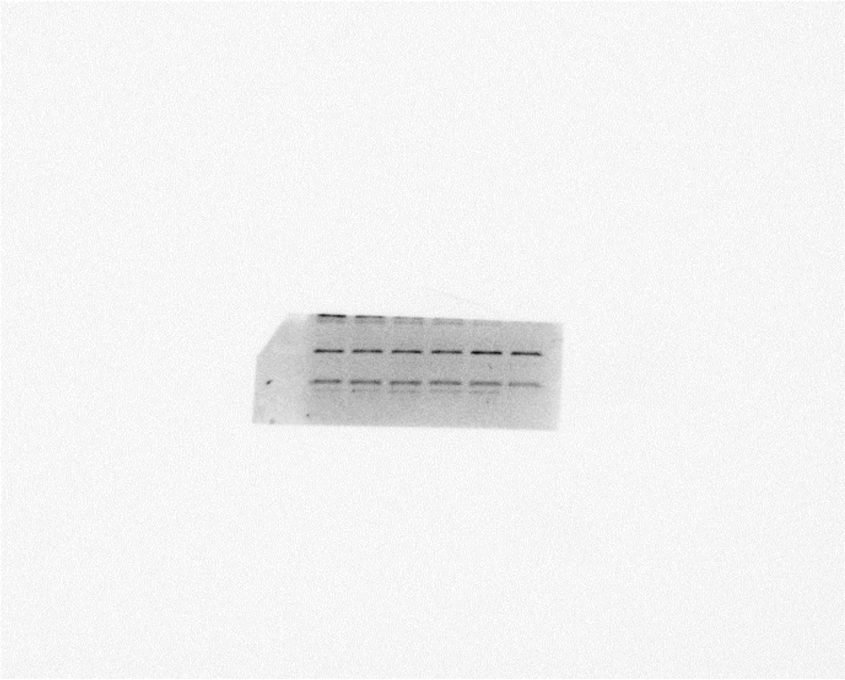

β-actin (0,1,2.5,5,10,20 μg/mL COP)

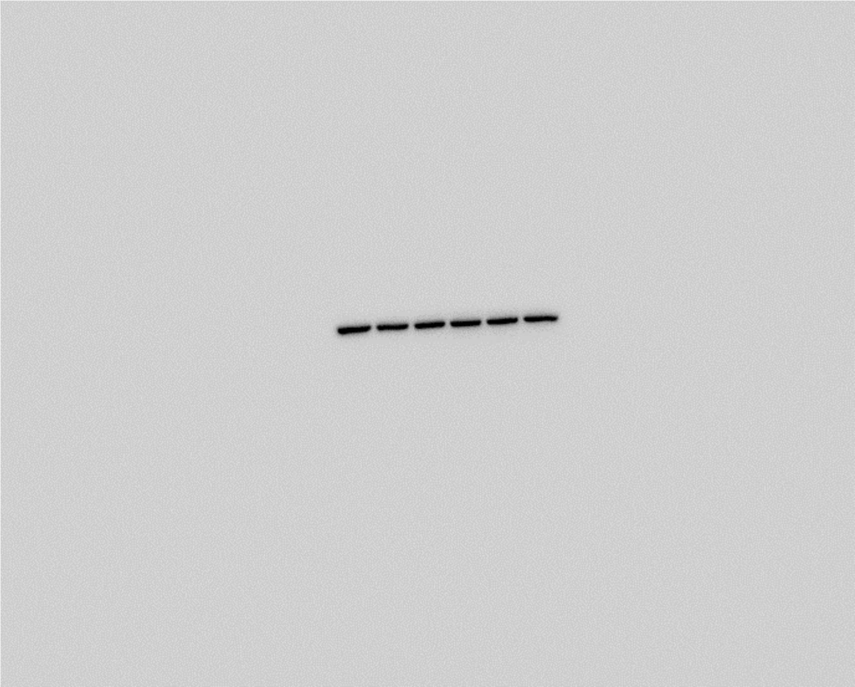

PI3K (0,1,2.5,5,10,20 μg/mL COP)

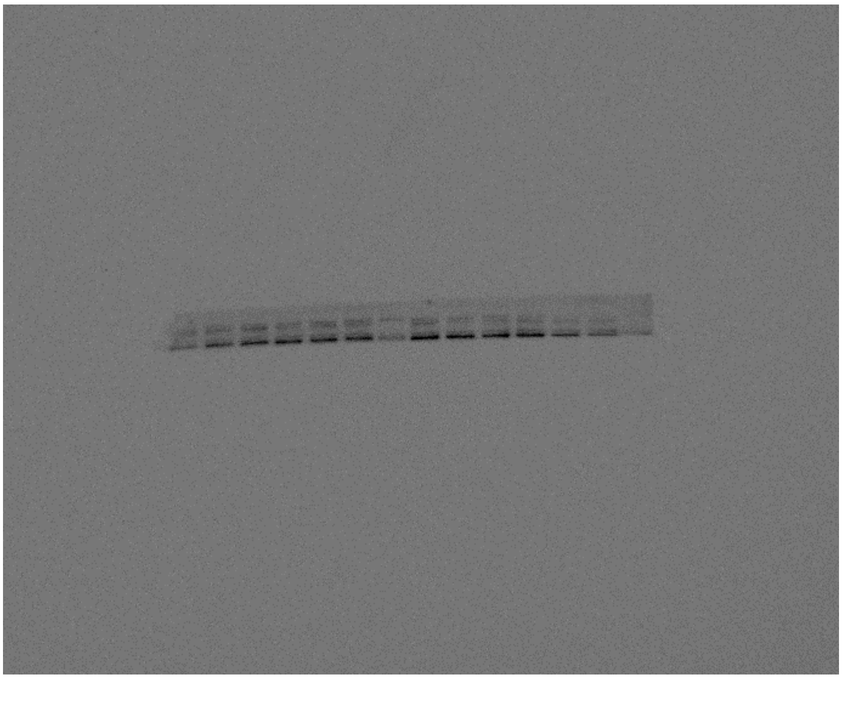

Akt (0,1,2.5,5,10 μg/mL COP)

p-Akt (0,1,2.5,5,10 μg/mL COP)

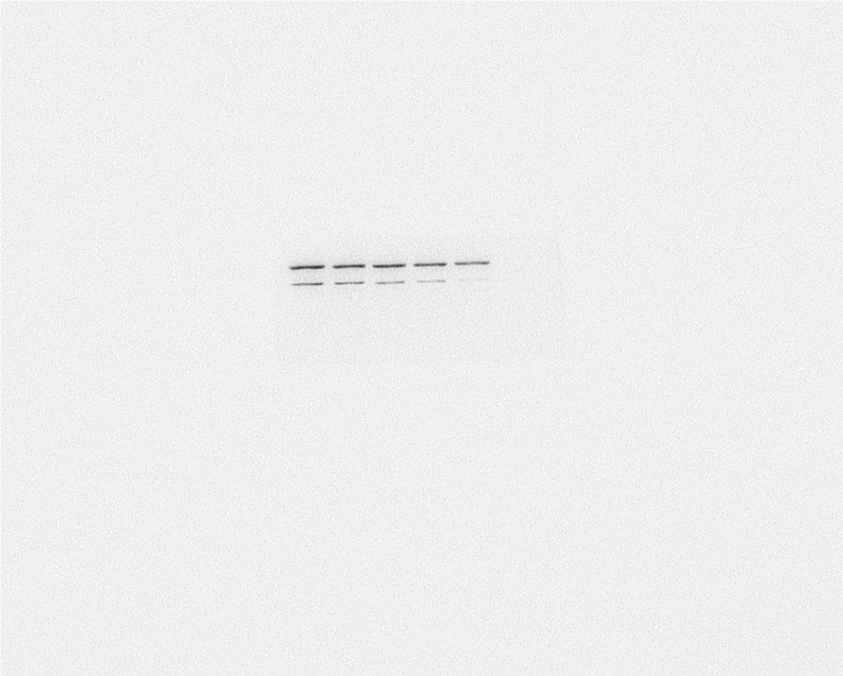

ERK (0,1,2.5,5,10 μg/mL COP)

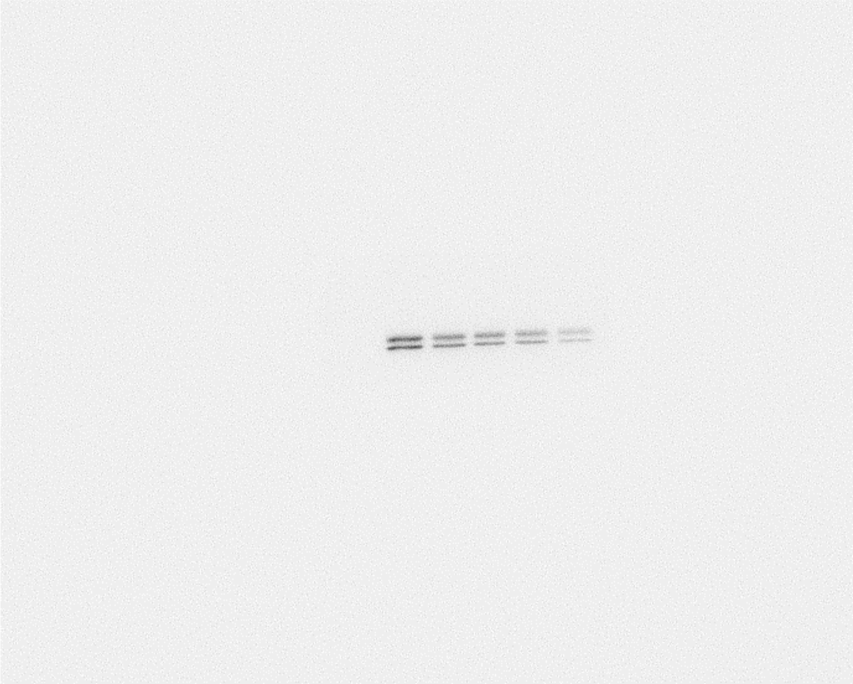

p-ERK (0,1,2.5,5,10 μg/mL COP)

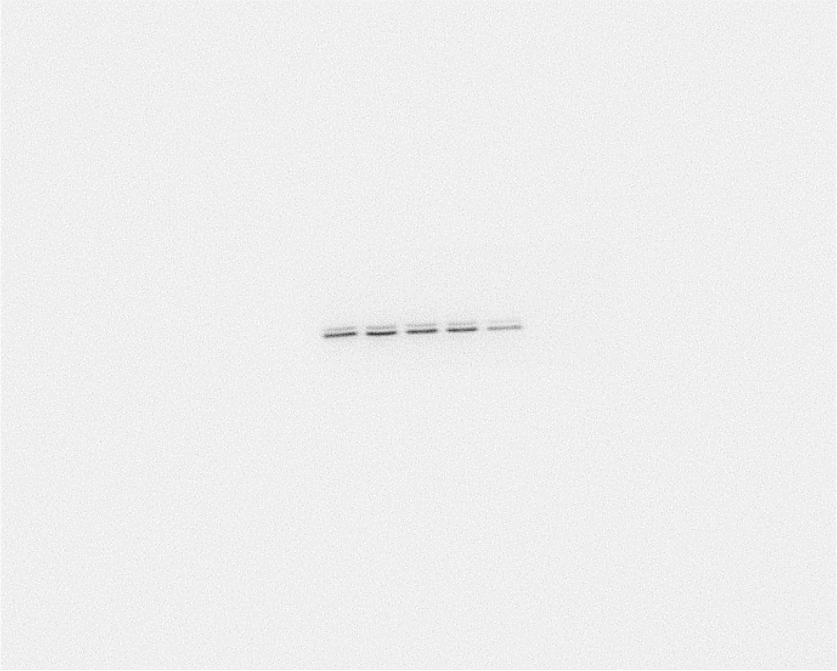

β-actin (0,1,2.5,5,10 μg/mL COP)

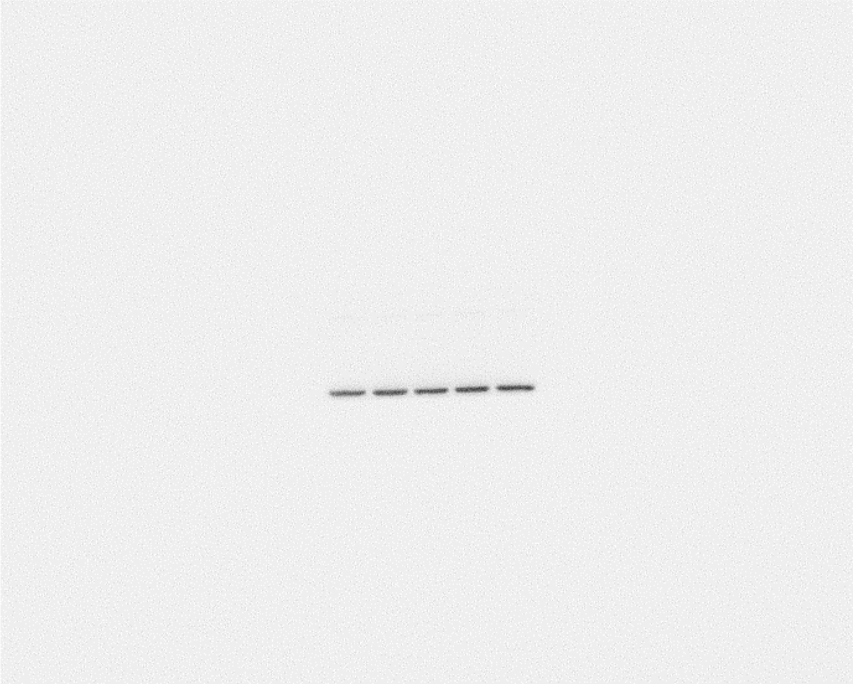

Cyclin D1 (0,1,2.5,5,10 μg/mL COP)

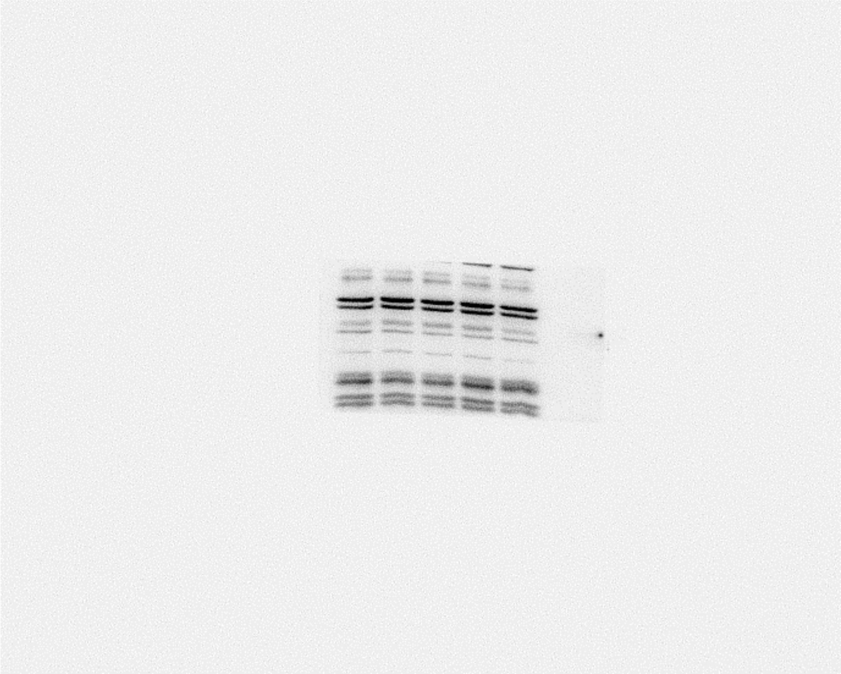

CDK4 (0,1,2.5,5,10 μg/mL COP)

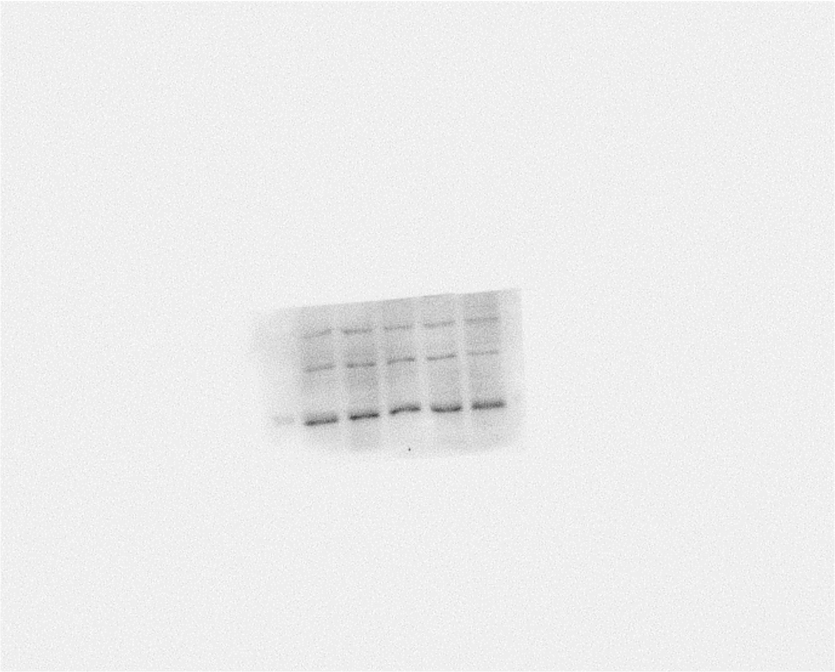

Cyclin E1 (0,1,2.5,5,10,20 μg/mL COP)

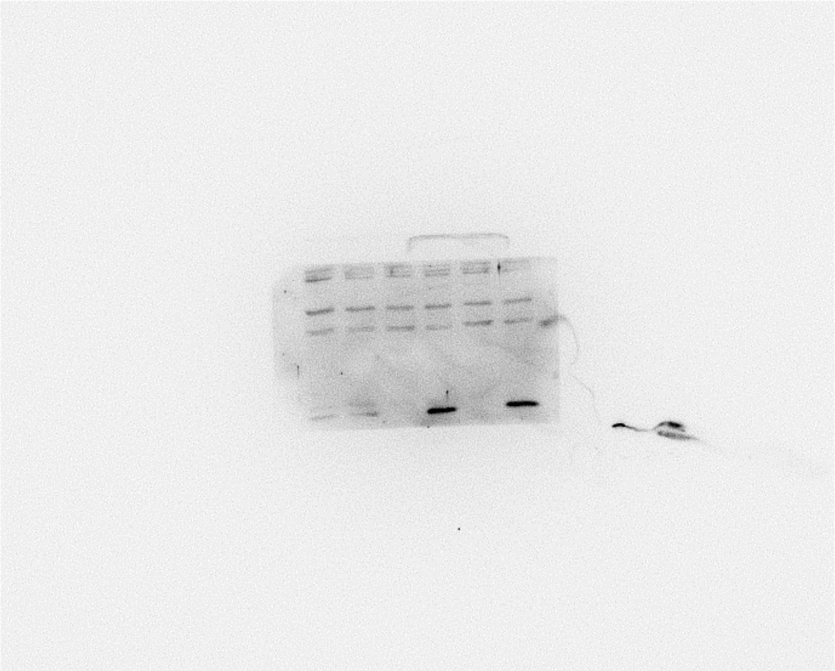

CDK2 (0,1,2.5,5,10μg/mL COP)

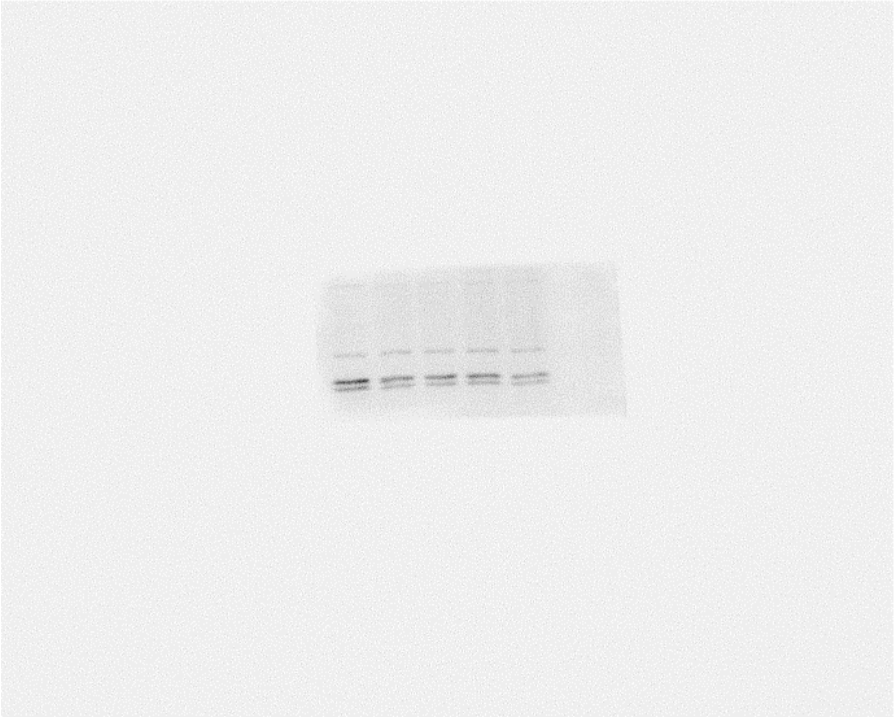

β-actin (0,1,2.5,5,10 μg/mL COP)

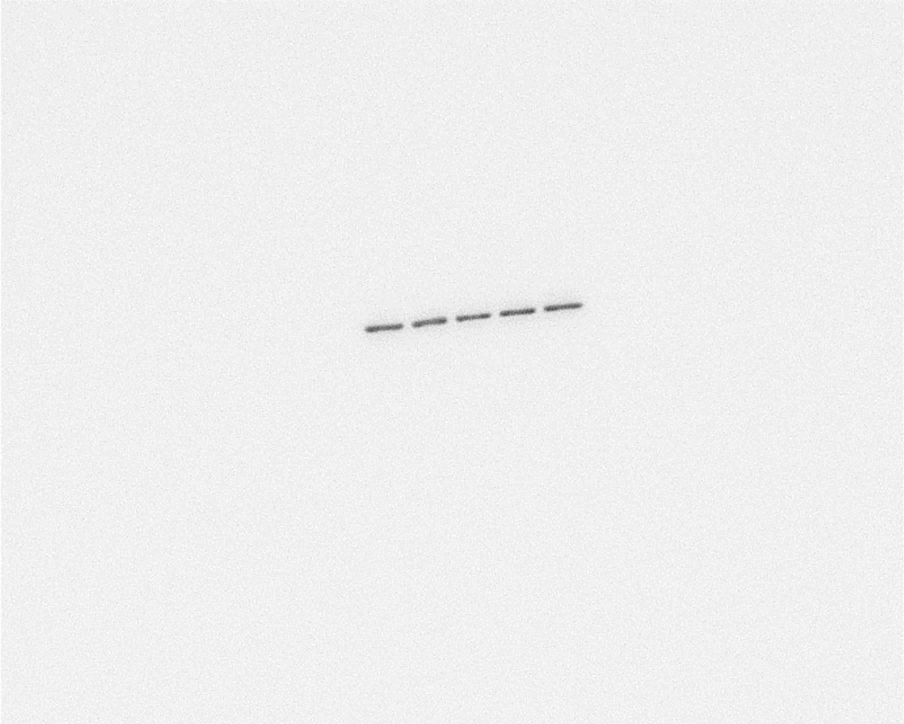

Supplementary Figure and Figure legends

Figure S1

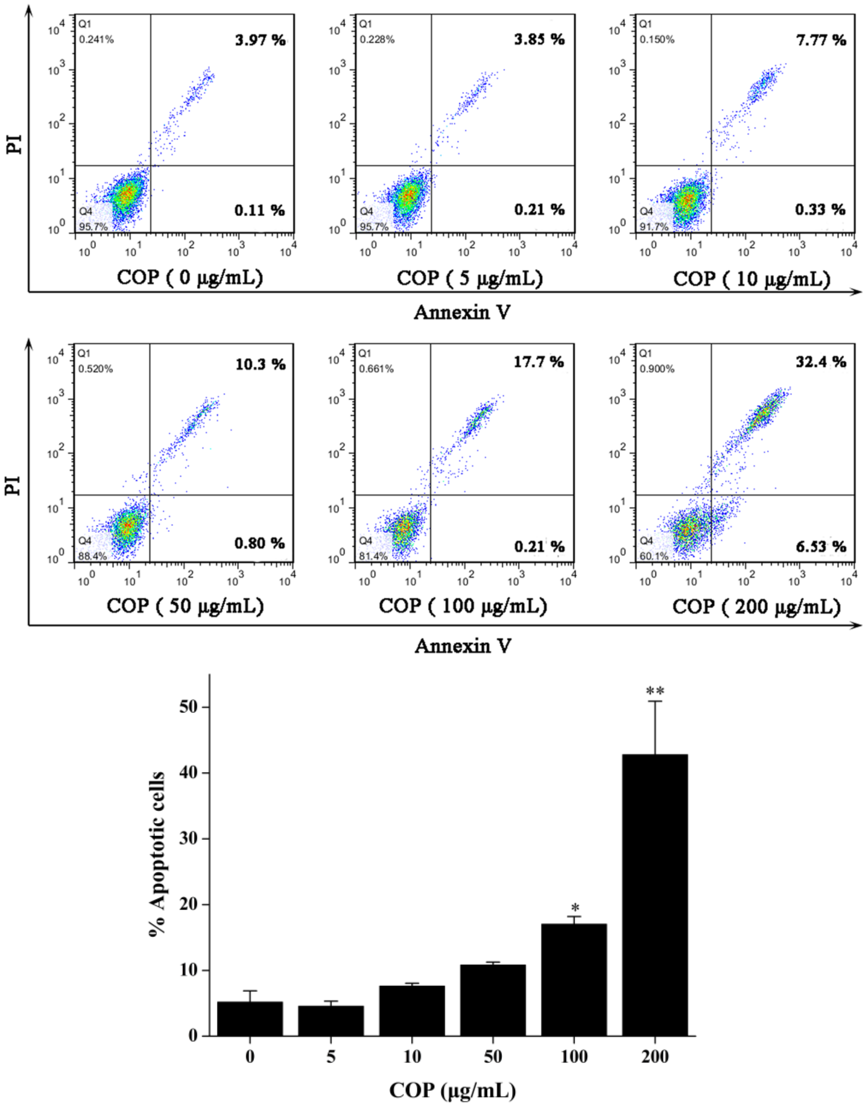

Figure S1. Apoptosis occurred in FHC after COP treatment. Cells were treated with indicated concentrations of COP for 24 h. Apoptotic cells were quantified by flow cytometry after stained with Annexin V and PI. The data represented were mean percentage ± SD of three replicates (*P<0.05, **p < 0.01 vs control group).

Figure S2

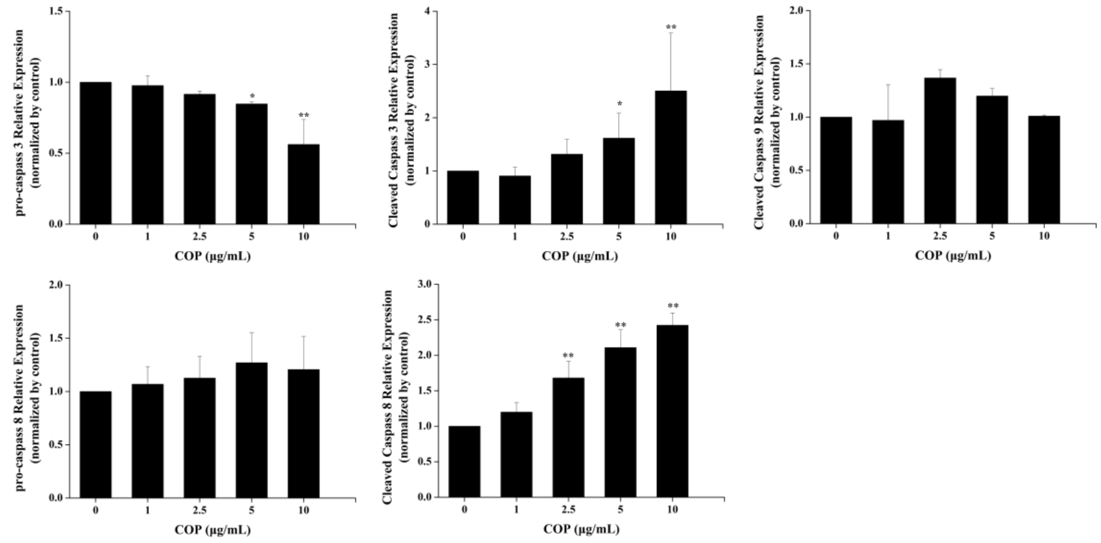

Figure S2. Effect of COP on HCT-116 apoptosis related proteins pro-caspase 3 and 8, cleaved caspase 3, 8 and 9. Expression of proteins were determined by Western blotting. Data were expressed as mean relative amount ± SD of three replicates (*$p < 0.05$, **$p < 0.01$ vs control group).

Figure S3

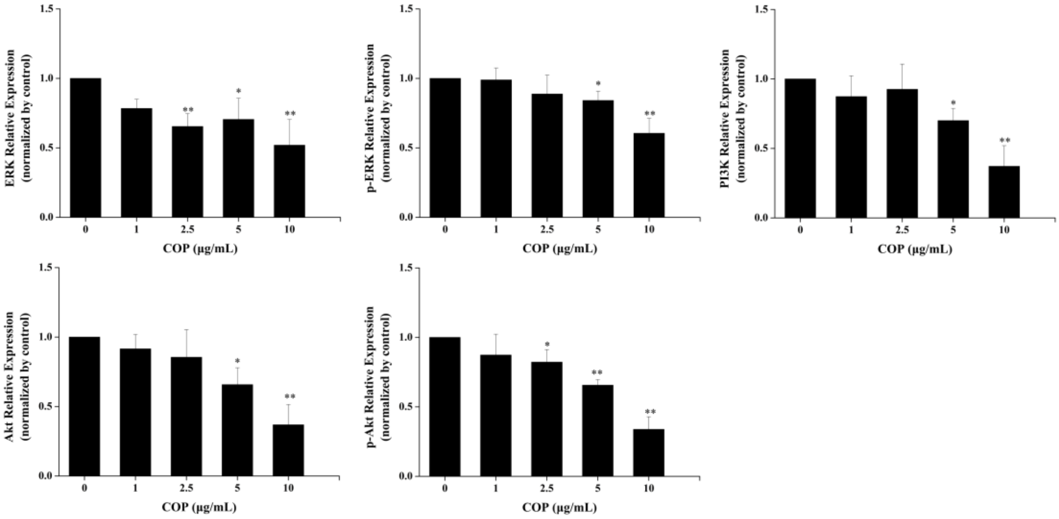

Figure S3. Effect of COP on HCT-116 survival related proteins ERK, p-ERK, Akt, p-Akt and PI3K. Expression of proteins were determined by Western blotting. Data were expressed as mean relative amount ± SD of three replicates (*p< 0.05, **p < 0.01 vs control group).

Figure S4

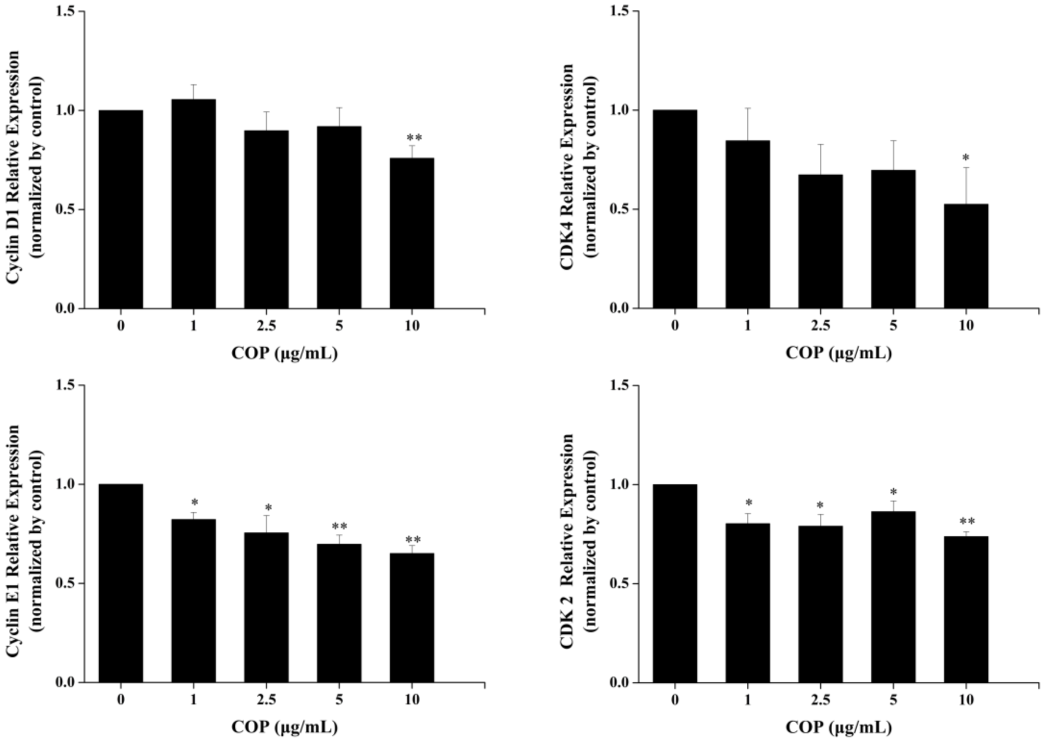

Figure S4. Effect of COP on HCT-116 cell cycle related proteins Cyclin D1, CDK4, Cyclin E1 and CDK 2. Expression of proteins were determined by Western blotting. Data were expressed as mean relative amount ± SD of three replicates (*$p < 0.05$, **$p < 0.01$ vs control group).

Figure S5

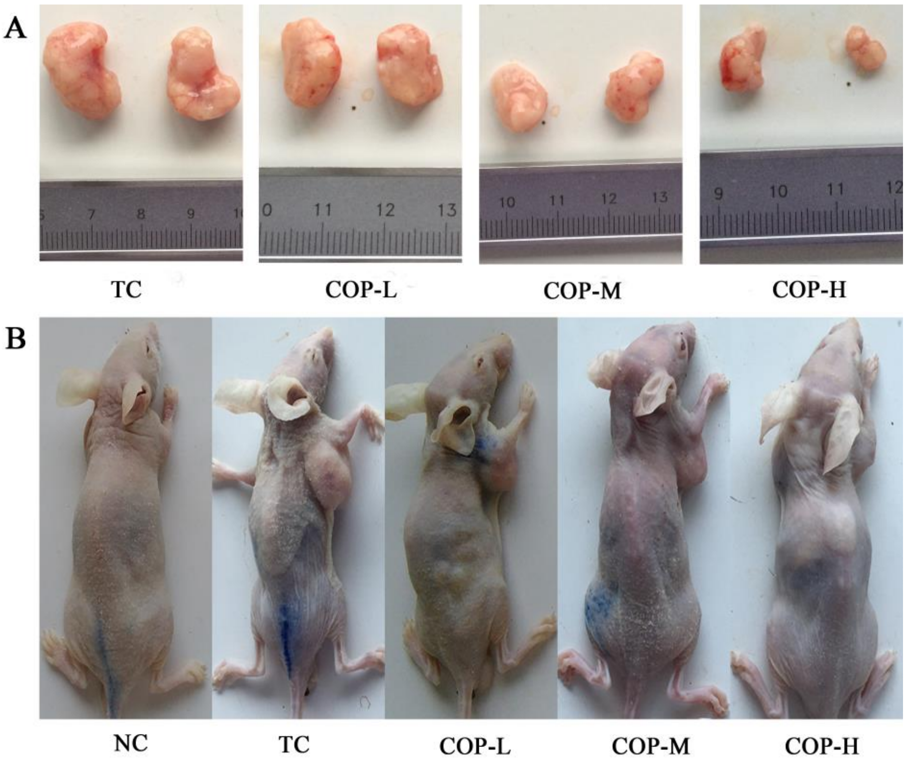

Figure S5. Effect of COP on tumor growth in human tumor xenograft mice. Representative photographs after treatment with COP and PBS were shown. (A) the tumors. (B) the whole animal. NC represented negative control; TC represented tumor control. COP-L: Coptisine at low dosage (50 mg/kg); COP-M: Coptisine at medium dosage (100 mg/kg); COP-H: Coptisine at high dosage (150 mg/kg).

Figure S6

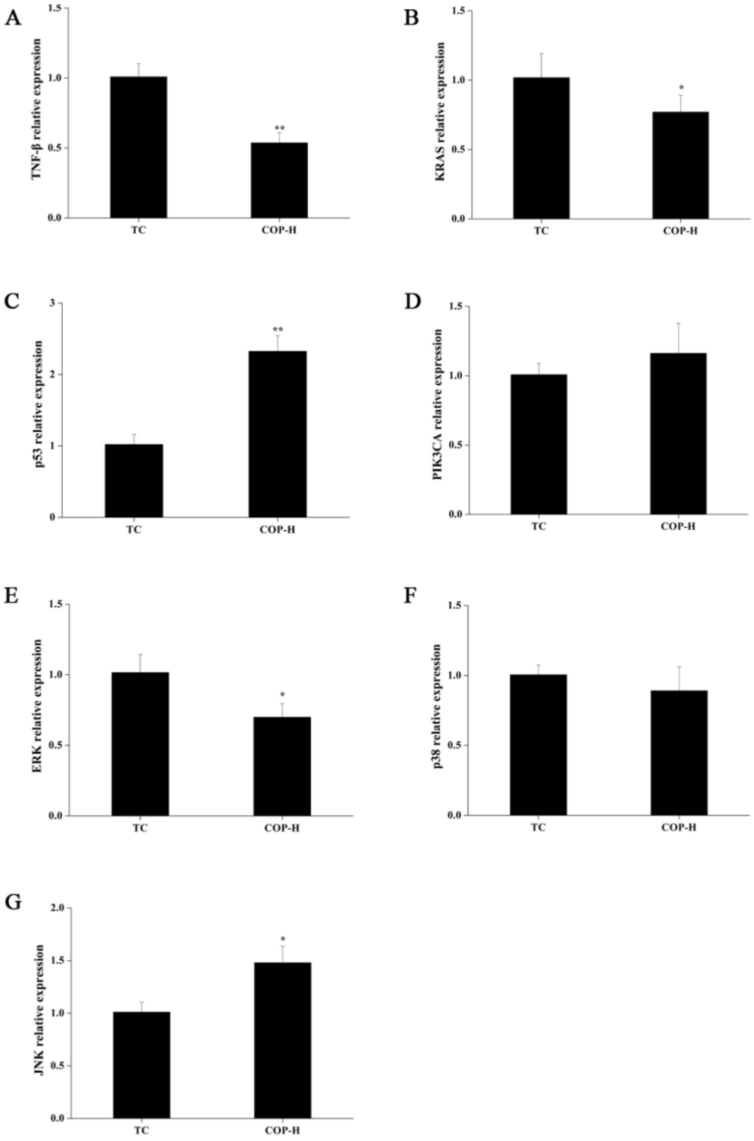

Figure S6. COP effected tumor MAPK pathway expression. The xenograft mice were administered with indicated concentrations of COP for 25 days. At the time of sacrificing, the tumors were obtained and extracted RNA. Effects of COP on MAPK pathway were assessed by qRT-PCR. The data were represented as relative mRNA levels for (A) TNF-β, (B) KRAS, (C) p53, (D) PIK3CA, (E) ERK, (F) p38 and (G) JNK. All values were expressed as mean ± SD ($n = 10$, *$p < 0.05$, **$p < 0.01$ vs TC group). TC represented tumor control. COP-H: Coptisine at high dosage (150 mg/kg).

Figure S7

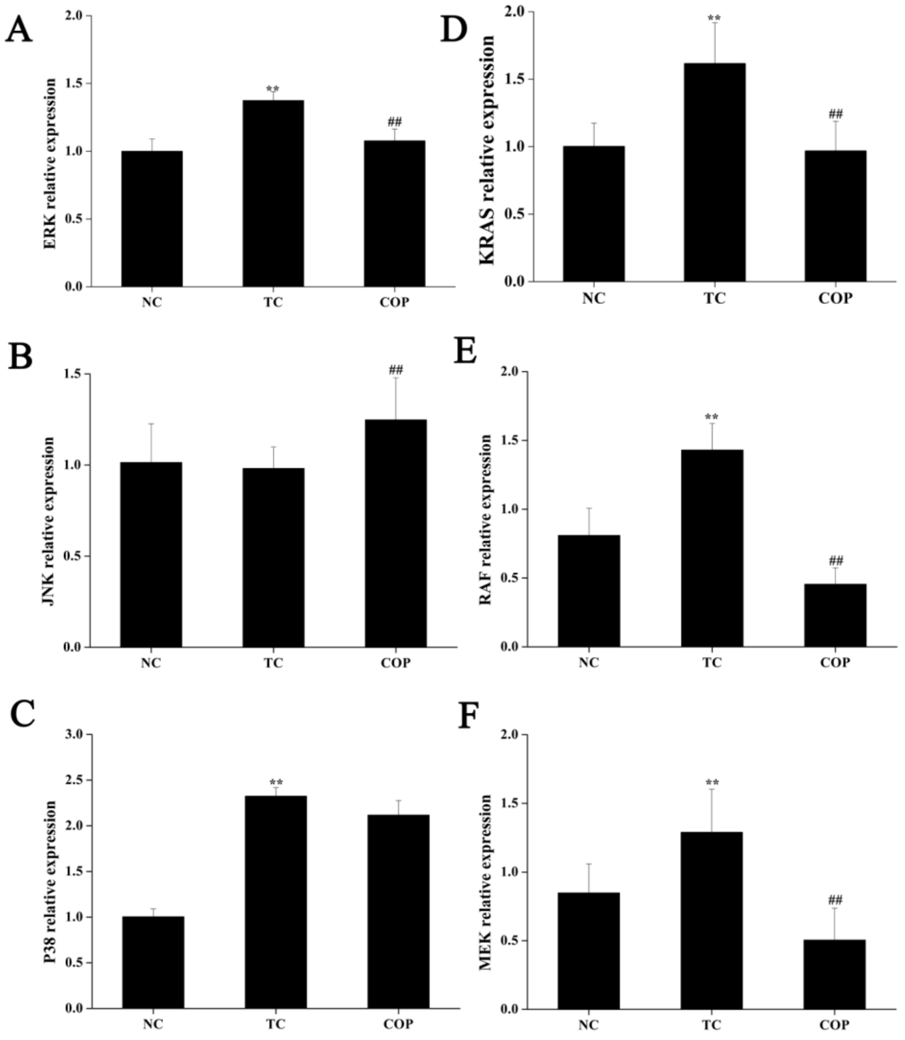

Figure S7. COP effected lung MAPK pathway and RAS-ERK pathway expression. The xenograft mice were administered with indicated concentrations of COP for 25 days. At the time of sacrificing, the lungs were obtained and extracted RNA. Effects of COP on MAPK and RAS-ERK pathway were assessed by qRT-PCR. The data were represented as relative mRNA levels for (A) ERK, (B) JNK, (C) p38, (D) KRAS, (E) RAF and (F) MEK. All values were expressed as mean ± SD (n = 10, *p < 0.05, **p < 0.01 vs NC group, #p < 0.05, ##p < 0.01 vs TC group). NC represented negative control; TC represented tumor control; COP represented coptisine at high dosage (150 mg/kg).
